# Children eat their school lunch too quickly: an exploratory study of the effect on food intake

**DOI:** 10.1186/1471-2458-12-351

**Published:** 2012-05-14

**Authors:** Modjtaba Zandian, Ioannis Ioakimidis, Jakob Bergström, Ulf Brodin, Cecilia Bergh, Michael Leon, Julian Shield, Per Södersten

**Affiliations:** 1 Karolinska Institutet, Section of Applied Neuroendocrinology, NVS, Mandometer and Mandolean Clinics, S-141 04, Huddinge, Sweden; 2 Karolinska Institutet, Medical Statistics Unit, S-171 77, Stockholm, Sweden; 3 University of Bristol & Bristol Royal Hospital for Children, Upper Maudlin Street, Bristol, BS2 8AE, UK

## Abstract

**Background:**

Speed of eating, an important aspect of eating behaviour, has recently been related to loss of control of food intake and obesity. Very little time is allocated for lunch at school and thus children may consume food more quickly and food intake may therefore be affected. Study 1 measured the time spent eating lunch in a large group of students eating together for school meals. Study 2 measured the speed of eating and the amount of food eaten in individual school children during normal school lunches and then examined the effect of experimentally increasing or decreasing the speed of eating on total food intake.

**Methods:**

The time spent eating lunch was measured with a stop watch in 100 children in secondary school. A more detailed study of eating behaviour was then undertaken in 30 secondary school children (18 girls). The amount of food eaten at lunch was recorded by a hidden scale when the children ate amongst their peers and by a scale connected to a computer when they ate individually. When eating individually, feedback on how quickly to eat was visible on the computer screen. The speed of eating could therefore be increased or decreased experimentally using this visual feedback and the total amount of food eaten measured.

**Results:**

In general, the children spent very little time eating their lunch. The 100 children in Study 1 spent on average (SD) just 7 (0.8) minutes eating lunch. The girls in Study 2 consumed their lunch in 5.6 (1.2) minutes and the boys ate theirs in only 6.8 (1.3) minutes. Eating with peers markedly distorted the amount of food eaten for lunch; only two girls and one boy maintained their food intake at the level observed when the children ate individually without external influences (258 (38) g in girls and 289 (73) g in boys). Nine girls ate on average 33% less food and seven girls ate 23% more food whilst the remaining boys ate 26% more food. The average speed of eating during school lunches amongst groups increased to 183 (53)% in the girls and to 166 (47)% in the boys compared to the speed of eating in the unrestricted condition. These apparent changes in food intake during school lunches could be replicated by experimentally increasing the speed of eating when the children were eating individually.

**Conclusions:**

If insufficient time is allocated for consuming school lunches, compensatory increased speed of eating puts children at risk of losing control over food intake and in many cases over-eating. Public health initiatives to increase the time available for school meals might prove a relatively easy way to reduce excess food intake at school and enable children to eat more healthily.

## Background

While nutritional standards for setting down what school children should eat for school meals have been recommended e.g., [1], there are no studies on eating behaviour during school lunches. The pattern of eating behaviour may be important in determining how much food is consumed. For example, individuals can be divided into those who eat at a decelerating speed (decelerated eaters) and those eating at a nearly constant speed (linear eaters) over the course of a meal [2]. Whilst decelerated eaters seem able to regulate their food intake, linear eaters eat too much when forced to eat quickly and too little when having to eat slowly [2,3]. Linear eating may therefore be a behavioural risk factor for loss of control over food intake. In fact, linear eaters overeat and adopt the eating behaviour of obese patients when the speed of eating is experimentally increased; at the same time, their estimation of satiety (fullness) post consumption decreases [4]. In addition, a high speed of eating characterises obese patients [4] and slowing the speed of eating has been successfully used in reducing meal size and body weight whilst improving key hormonal responses to oral glucose in obese children and adolescents [5,6].

Thus, the pattern and speed of eating can influence total food intake; in particular, an increased speed of eating may put individuals at risk of eating too much. Children at school may be exposed to this risk because the time available for the school lunch is very brief [7-10]. Hence, school children may be forced into eating quickly, possibly losing control over food intake. We have explored if this is the case and examined how experimental variations in the speed of eating affect food intake in a sample of school children.

### Study 1: time spent eating the school lunch

We first measured the time spent eating lunch in a large group of students, which has been done before in the United States [7,8]. This was a descriptive study, no hypothesis was tested.

## Methods

### Participants

Ten out of the 22 secondary schools in Stockholm were randomly selected. Five girls and five boys, aged 11–13, were studied in each of the 10 schools. Hence a total of 100 students were recorded.

### Ethics

In each school, the dean was first informed that the aim of the study was to measure the time spent eating amongst the students. Both teachers and students were then informed of the study’s aim and that they could decline from participating without giving a reason. The procedures were approved by the Central Ethical Review Board in Stockholm.

### Procedure

The children came to lunch between 11.30 am and 12.00 pm and the time spent waiting to be served, in other activities, e.g., talking to their peers, and in eating was measured using stop watches by two researchers, who were hidden from the students. The time spent eating was defined as the time from the first to the last bite. The children came in groups of 3–5. Boys were served before girls and, for this reason, the five girls and the five boys who came to lunch first were studied. Using three stop watches each, the two researchers recorded 10 students (5 girls), from each of the 10 schools. Results were obtained from a total of 100 students.

**Figure 1:**
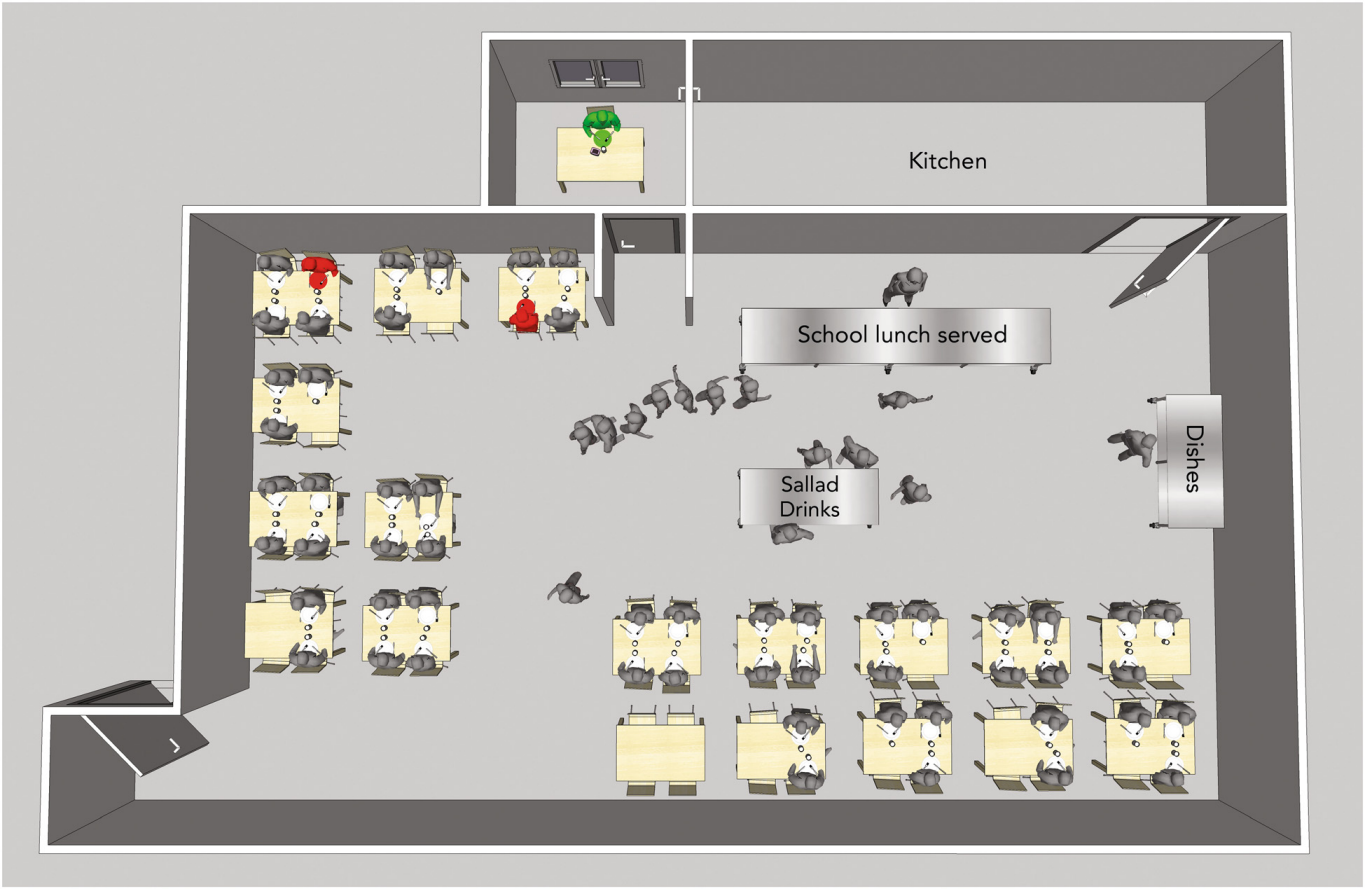
**School lunch room.** The children ate their lunch on a hidden scale (red) together with the other children or using Mandometer® individually (green).

The lunch rooms had an outline similar to that shown in Figure 1.

## Results

The lunch break lasted on average (SD) 46.5 (7.5) minutes; the children came in groups of least 5–10 and at most 20–30 to the lunch room.

The 100 children spent 13.7 (1.3) minutes from entering to exiting the lunch room, but they spent only 7 (0.8) minutes eating. The other time was spent waiting to be served (4 (0.9) minutes) and talking to the other students (2.1 (0.8) minutes). There was no gender variation in these measures, which were therefore combined.

Very little time was spent talking when the children were eating: talking amongst students occurred when they were waiting to be served. The time spent in the lunch room, 13.7 minutes, was longer than the combined time spent waiting, talking and eating, 13.1 minutes, because when the children had finished eating they returned their food tray to the dishes.

### Study 2: speed of eating and food intake

Having confirmed that school children ate their lunch in a short period of time [7,8], we examined how this affected their eating behaviour and their food intake by experimentally increasing or decreasing the speed of eating when the children ate individually, a question that has not been addressed before. This was an experimental study, testing the hypothesis that a change in the speed of eating affects food intake. Interestingly, it has been suggested that an increased speed of eating is a cause of overeating in obesity [4-6] and it has been shown that a decreased speed of eating decreases food intake in linear eaters [2].

## Methods

### Participants

A secondary school in the Stockholm area was randomly selected. The school had 800 students, 275 were between 12–15 years old, two classes had 55 students who were 13 years old and these students were approached. The students were recruited by an advertisement close to the lunch room. 18 girls participated; their mean (SD) age was 13.1 (0.4) years and their body mass index (BMI, weight/height squared) was 19.4 (0.9) kg/m^2^. 12 boys took also part; their age was 13 (0.5) and their BMI was 20.1 (0.8). The children were healthy and had no eating disorder symptoms as determined by a questionnaire [11].

### Ethics

The children were informed verbally and their parents were informed in a letter that the goal of the study was to examine how much students ate at lunch. The children could leave the study at any time without giving a reason. Parents and children gave written consent to participate. The procedures were approved by the Central Ethical Review Board in Stockholm.

### Apparatus and experimental manipulation of the speed of eating

Mandometer® is a weighting scale and a custom-made computer with a 15 in. touch screen (Mikrodidakt AB, Lund, Sweden). The computer stores the weight loss data generated when a subject eats food from a plate placed on the scale and the cumulative food intake is modelled by: y = kx^2^ + lx, where the k-coefficient reflects the change in eating speed over time, i.e., the degree of deceleration of the eating rate over the course of the meal, and the l-coefficient reflects the initial speed of eating [2].

Once the curve of the cumulative food intake has been determined for an individual, her or his eating behaviour can be experimentally modified. This is achieved by programming the computer with the amount of food to be consumed and the duration of the meal; the software calculates the k- and l-coefficients, and the corresponding cumulative intake curve can be displayed on the computer screen. Such experimentally changed curves are used as feedback guiding the individual to eat in a predetermined manner. For the purpose of testing the present hypothesis that a change in the speed of eating affects food intake, the cumulative curve of food intake that each individual student generated when eating individually in unrestricted conditions was displayed on the computer screen, but the duration of the meal was shortened or prolonged (see below). The subject can adjust her/his speed of eating to these feedback curves because her/his own speed of eating emerges on the computer screen during the meal and can therefore be compared to the reference, feedback curve.

The subject estimates her/his feeling of fullness (satiety) from nothing at all (0) to maximal (10) on a scale, which also appears on the computer screen during the meal. Similar methods have been used before [12-14], Mandometer® adds the possibility of experimentally controlling food intake [2,4]. A brief video shows how it works [15].

### Procedure

35 min were allocated for lunch served between 10.50 am and 12.40 pm in a room, about 90 m^2^ in size, where 72 students could eat at a time (Figure 1). The children were served at 11.30 am and at 12.00 pm. In the experimental meals, they were tested individually in a separate room, 6 m^2^ in size (Figure 1); they came 10 min before the meal and relaxed for another 10 min after the meal. The food (rice, sliced chicken and vegetables, Findus, Bjuv, Sweden; 400 kJ, 4.5 g protein, 18 g fat and 15 g carbohydrate/100 g) was prepared fresh before each meal. The children were instructed to eat as much as they wanted and to estimate their satiety before and after the meal. The meal started when the child took the first mouthful of food from the plate on the scale of the Mandometer® and it ended when the child had removed the last amount of food from the plate, put it into her/his mouth, chewed and swallowed the food and pressed a button on the Mandometer® screen that s/he had finished eating.

### Testing conditions

To get accustomed to the experimental method, the children first ate using Mandometer®, with no data collection. Participants are easily familiarized with the use of Mandometer® including how to adjust their eating to the curves which are displayed on the computer screen and used as feedback on how to eat. In several previous studies we have found that one session is sufficient to instruct study participants on Mandometer® usage.

The children were then tested under the following conditions.

1. ***Unrestricted meal:*** The children were first tested individually using Mandometer® with no feedback on the screen to determine their typical curve of cumulative food intake: henceforth referred to as the control meal. In this test, the children served themselves food from a serving dish with 1200 g food placed adjacent to the Mandometer®.

2. ***The school lunch:*** The children waited to be served with the other students, and they were instructed to eat at one of two tables with a hidden scale, built into the table, which was indistinguishable from the other tables (Figure 1). Four children ate at each table, the hidden scale was placed under the lower left quadrant of the table. The amount of food consumed was recorded by the scale and the duration of the meal was measured using stop watches as described above.

3. ***Experimental change of eating speed:*** The children ate individually using Mandometer® following the curve of cumulative food intake that each child had generated in the control meal, which was displayed on the screen. However, the time of the meal was increased or decreased by 30% or unchanged compared to the control meal. This was achieved by “compressing” the time, i.e., the scale of the x-axis on the Mandometer® to fit the same amount of food within a shorter or longer period of time. There was, however, no visual difference in the feedback provided on the screen. The amount of food that each child had eaten in the control meal was placed on the plate at the start of the experimental meals and the serving dish with additional food was available simultaneously.

The school lunch and the meals in which the speed of eating was experimentally changed were given in random order. Four children were tested each day, two at 11.30 and two at 12.00 am. All tests were separated by a week.

Satiety was recorded only before and after the meal. It is sufficient to test subjects once; the test-to-test variability is low [2].

### Statistical analyses

**Figure 2:**
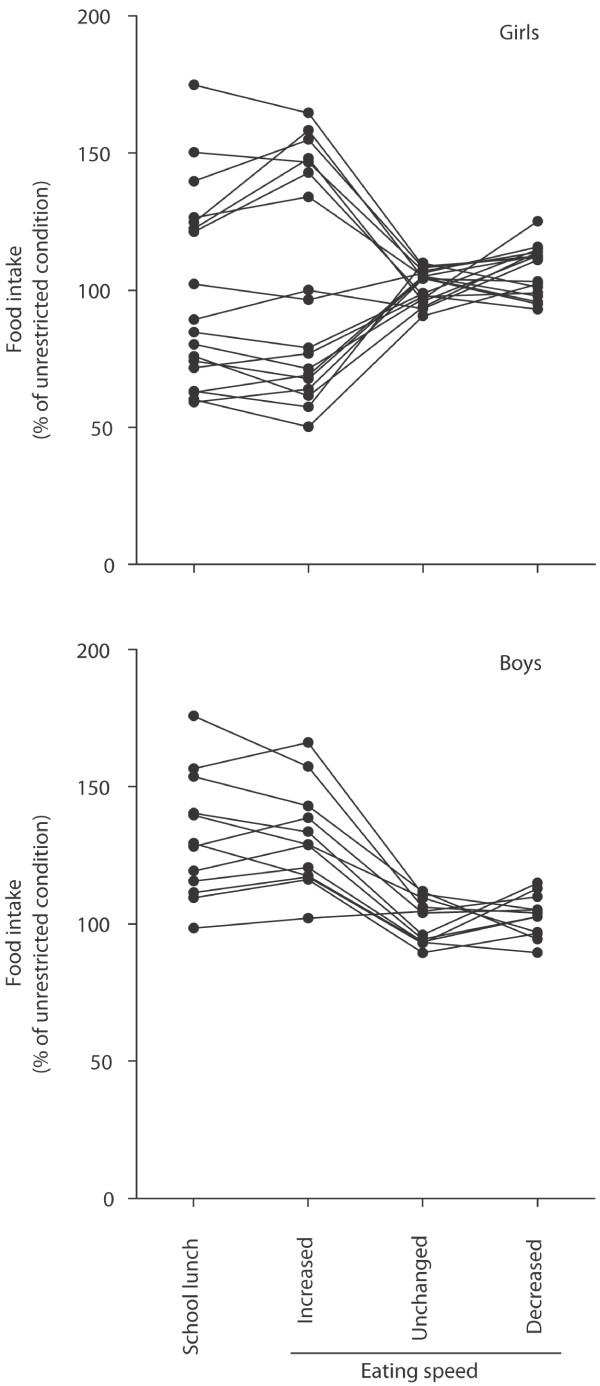
**Food intake in children in secondary school.** Food intake during the school lunch in girls (n = 18) and boys (n = 12). The children had their speed of eating experimentally increased, unchanged or decreased in relation to its value in a meal eaten without restrictions; values are expressed as percent of that value.

Data on the amount of food ingested, the duration of the meal and the level of satiety are expressed as mean (SD), (95% CI) or (range). Some children took more food when eating at an increased speed compared to when they ate their control meal thus increasing the total food intake. To facilitate data interpretation, the change in food intake in the school lunch and the experimental tests is expressed as percent of the intake in the control meal in Figure 2. Gender differences in eating behaviour were compared with t-tests. The variance in the food ingested was analyzed using mixed linear models and gender differences were estimated by the restricted maximum likelihood method [16].

Analyzes were performed using SAS 9.2 (SAS Institute Inc., Cary, NC, USA) and Statistica 8.0 (StatSoft Inc., Tulsa, OK, USA).

## Results

### Control meal

The first aim of Study 2 was to describe eating behaviour in girls and boys eating individually without constraints. It was found that girls and boys consumed about the same amount of food but girls took more time to eat and started eating with a lower speed than boys. The speed of eating changed over the course of the meal at a similar, decelerated rate in both sexes and the estimation of satiety before and after the meal was about the same (Table 1).

**Table 1:** **Eating behaviour and satiety in girls and boys in secondary school tested individually**^**a**^

**Characteristic**	**Girls**	**Boys**	**Difference (95% CI)**	***P***^**b**^
N	18	12		
Food intake (g)	258 (38)	289 (73)	-31.7 (−73.2 – 9.8)	.19
Meal duration (min)	10.7 (2.1)	8.8 (1.8)	1.8 (−0.3 – 3.3)	.02
Rate of deceleration^c^	−2.6 (1.4)	−2.7 (1.1)	0.1 (−0.9 – 1.1)	.82
Initial speed of eating				
(g/min)^d^	34 (5)	42 (7)	−8.8 (−13.4 –−4.3)	.002
Satiety (0–10):				
Before meal	1.2 (1.1)	1.3 (0.9)	−0.1 (−0.4 – 0.2)	.48
After meal	5.5 (0.8)	5.7 (1.2)	−0.2 (−0.9 – 0.5)	.6

^a^Values are mean (SD) or (95% CI).

^b^t-test.

^c^Rate of deceleration (k) in the model, y = kx^2^ + lx, of eating behavior.

^d^Initial speed of eating (l) in the model, y = kx^2^ + lx, of eating behavior.

### Time spent eating the school lunch versus eating individually

The second aim of Study 2 was to compare the time spent eating in girls and boys tested individually with the time spent eating their school lunch. It was found that both girls and boys finished their school lunch in a shorter period of time than the control meal (girls: mean: 5.6 (1.2) vs 10.7 (2.1) minutes, p < 0.001; boys: 6.8 (1.3) vs 8.8 (1.8) minutes, p = 0.003).

### Food intake in the school lunch and experimental meals

Having found that the children spent much less time eating their school lunch than when eating individually, the third aim of Study 2 was to examine how this difference affects food intake and how an experimental change in the speed of eating influences food intake. For this purpose, the food intake observed in the school lunch was compared to that observed when the children ate individually in the control condition and when the speed of eating was changed experimentally.

Food intake differed significantly in the five testing conditions (F(4,20) = 3.88, p = 0.017) and was significantly affected by gender (F(1,16) = 8.26, p = 0.011). There was a significant interaction between gender and testing condition (F(4,20) = 3.88, p = 0.017) (Table 2). The average food intake in the different conditions was similar amongst girls (Table 2), but there were marked individual differences.

**Table 2:** **Food intake (g) in girls and boys in secondary school tested in different experimental conditions**^**a**^

**Condition**	**Girls**	**Boys**	**Difference (95% CI)**	***P***^**b**^
N	18	12		
Control (Unrestricted^c^)	258 (38)	289 (73)	−31.7 (−73.2 – 9.8)	.13
School lunch^d^	251 (83)	372 (71)	−121 (−180 –−61)	< .001
Eating speed:				
Increased	257 (93)	372 (80)	−115 (−182 –−48)	.002
Unchanged	261 (36)	287 (58)	−26(−61 – 9)	.14
Decreased	276 (42)	300 (84)	−24 (−71 – 23)	.32

^a^Values are mean (SD) or (95% CI).

^b^t-test.

^c^The children ate the control meal individually in a separate room in relaxed conditions.

^d^The children ate the school lunch among the other children.

In reporting the findings, food intake in the different experimental conditions is expressed as percent of the intake in the individualised control meal for each child (see Figure 2).

Hence, the variation in food intake, rather than the amount of food eaten, was analysed as the main outcome of the experimental variation in the speed of eating. For this purpose a linear model assuming unequal variance at each time point and unequal dependency between time points was chosen (see Additional file 1).

During the school lunch, seven out of the 18 girls ate more (23%, range: 19–64) and nine ate less (33%, range: 17–51) food; only two maintained their intake at the control level (−3% and 0.5%) (Figure 2). This pattern of food intake could be replicated by experimentally increasing the speed of eating for an individual meal yielding a high correlation between food intake in these two conditions (r = 0.93). Experimentally decreasing the speed of eating or maintaining the control-level had no effect (Figure 2), yielding high correlations between food intake in these conditions (r = 0.85, and r = 0.91, respectively) (Figure 2). Conversely, there was no correlation between intake during the school lunch and when the speed of eating was experimentally decreased (r = 0.10) or unchanged (r = 0.06).

Food intake was less variable and highly correlated in all combinations of the testing conditions (range: r = 0.73-0.97) amongst boys (Figure 2). 11 of the 12 boys ate more food in the school lunch (26%, range: 9–60), only one maintained his intake at control levels (3%). This pattern of intake was replicated experimentally in boys as in girls (Figure 2).

The children estimated their satiety before and after the experimental meals as similar to their estimations in the control meal (Table 2) (data not shown).

It is noteworthy that although the girls ate about the same amount of food when they ate individually in the control setting as they did when they ate their school lunch together with the other students, they spent a shorter time eating. Thus, during the school lunch their speed of eating increased to 183% (range: 97–272) of the value recorded in the control meal. Equally noteworthy, the boys ate more at lunch than when they ate individually yet they spent much less time eating lunch. As a result, their speed of eating at lunch increased to 166% (range: 118–285) of the control meal.

## Discussion

The finding that the 100 children in Study 1 spent around seven minutes eating lunch is similar to that reported in previous studies, which, in addition reported that the time spent eating the school lunch does not vary very much with age [7,8,10,17]. Similarly, in Study 2, children spent very little time eating lunch, markedly increasing their speed of eating compared to when eating individually in the control setting. As a consequence, about half the girls ate more food, most of the other girls ate less food with only two maintaining their intake at the control level and all but one of the boys ate more food. Gender differences in food intake similar to that found in our study have been reported before in young children stressed to eat quickly [3,18]. These changes in food intake during school lunches were replicated by experimentally increasing the speed of eating. At the same time, the feeling of fullness was not affected, indicating that the perception of satiety does not assist in eating a proper amount of food when eating quickly [19]. Thus, the present findings differ from findings in adult humans and pre-school children that eating together with others increases food intake but does not affect the speed of eating, possibly because there were no time constraints in these studies [20,21].

During control meals, the speed of eating decreased over the course of the meal in a similar way in girls and boys. This decelerated pattern of eating decreases with age in girls, but not boys [3,14,18,22] and young women tend to eat with a constant speed, i.e., assuming a linear pattern of eating which puts them at risk of eating too much when challenged to eat quickly [2,4,23]. Being compelled to eat the school lunch too quickly may further increase this risk.

School lunches are “rushed” in many countries and our results suggest that this may cause an increase in the speed of eating thereby distorting food intake. Because these observations were made on a limited number of children, Study 2 should be considered exploratory. However, the hypothesis is plausible because the distortions in food intake during the school lunch were replicated experimentally in all children. If these results can be obtained in larger groups of children, they would have considerable implications for public health. While nutritional factors are obviously important [1], it should also be recognized that human eating behaviour is physiologically regulated only in conditions that are very different from those of modern life [24]. Today, eating is controlled more by the cost and availability of food than by internal mechanisms or rational thought [25,26]. Interestingly, the importance of environmental factors for the development of obesity, particularly in children, was recently pointed out [27]. If additional studies can further verify the hypothesis that a high speed of eating is the cause of distorted food intake, including obesity [5], policies to reduce the speed of eating amongst school children are indicated, including methods for creating a relaxed atmosphere during the lunch break.

It should be recognized that many factors influence food intake and eating behaviour in school children. These include skipping meals and breakfast, the characteristics of the individual child, the interaction between individuals, and the physical environment [28], and, unsurprisingly, the availability of high-caloric, easily consumed foods [29]. Interestingly, if easily available, these can actually increase intake when children eat individually [30]. These are some of the topics which need to addressed in future studies.

## Conclusions

We confirmed results from previous studies that school children spend very little time eating their lunch. We suggest that currently, school lunches favour increased speed of eating which influences total food intake during the meal. Whilst half of the females respond by decreasing total food intake, 40% increase as do the vast majority of boys. The conditions at the school lunch should be adapted to allow children to eat a normal amount of food at a normal speed.

### Limitations

The main limitation of this study is the relatively small number of children assessed, and thus this study should be considered exploratory. However, the high correlation between food intake in the school lunch and food intake in the test when the speed of eating was experimentally increased provides compelling preliminary evidence that the rapid consumptions of food during school lunches is deleterious to healthy eating behaviours.

## Competing interests

C Bergh and P Södersten each have 27.50% stock in Mando Group AB and M Leon has 3%. Mandometer AB, a fully owned subsidiary to Mando Group AB, holds the intellectual property rights to Mandometer®.

## Authors’ contributions

MZ, II, CB and PS participated in the planning of this study, MZ performed the studies, all authors took part in analyzing the results and writing the manuscript. All authors read and approved the final manuscript.

## Pre-publication history

The pre-publication history for this paper can be accessed here:

http://www.biomedcentral.com/1471-2458/12/351/prepub

## Supplementary Material

Additional file 1 Statistical analysis. Click here for file
