# Supplementary material for: Children eat their school lunch too quickly: an exploratory study of the effect on food intake
Source: BMC Public Health. 2012 May 14;12:351. doi: 10.1186/1471-2458-12-351 (PMC3490778; doi:10.1186/1471-2458-12-351)
Supplement: Additional file 1 — Statistical analysis. [file 1471-2458-12-351-S1.pdf]

Additional file 1 – Statistical analysis

As opposed to conventional analysis of variance, the use of mixed linear models makes it possible to estimate variances and covariances with a minimum of constraints. Model fit was evaluated for six models with different parameter constraints using -2 log restricted maximum likelihood (-2 log REML) and Akaike's information criterion [1]. Chi^2-tests were used for comparing smaller models with larger ones. The most constrained model (compound symmetry) assumed equal covariance (dependency) between all pairs of experimental conditions, a common variance for each experimental condition. This structure was assumed to be the same for girls and boys. The least constrained model (unstructured) assumed separate variances for each condition and different covariances (dependency) between pairs of conditions. This structure was estimated separately for girls and boys. The fitted models included sex and condition as independent variables, as well as an interaction term. In case of a significant interaction, simple main effects were analyzed. Cook's distance was used to reveal influential observations on the estimated model parameters [1]. Normal probability plots were used to evaluate whether the normal assumption was satisfied. All tests were two-tailed. Spearman correlation coefficients were used to correlate food intake in different experimental conditions.

Two linear models, both assuming unequal variance at each time point and unequal dependency between time points but with separate or equal variance estimates for girls and boys yielded the same goodness of fit (Table 1). The former model was chosen.

Table 1. Selection of covariance model. The structures are ordered from worst to best

Covariance structure	for sex	-2 log REML[a]	P[b]	AIC[c]	df
Symmetry[d]	Same	1550	NA[e]	1554	2
Symmetry[d]	Different	1527	<0.001	1535	4
Heterogeneity[f]	Same	1522	0.082	1534	6
Heterogeneity[f]	Different	1488	<0.001	1512	12
Unstructured[g]	Same	1407	<0.001	1437	15
Unstructured[g]	Different	1377	0.012	1437	30

a: -2 log restricted maximum likelihood

b: Chi^2-test

c: Akaike's information criterion

d: Equal variance in each condition and equal dependency between pairs of experimental conditions

e: Not applicable

f: Estimated variance for each conditions and equal dependency between pairs of experimental conditions

g: Estimated variance for each condition and estimated dependency between each pair of experimental conditions

Reference

1. Brown H, Prescott R. *Applied Mixed Models in Medicine.* Chichester: John Wiley & Sons Ltd 2006:218-226
